# Prognostic associations of vitamin D deficiency with disease severity, survival, and complications in alcohol-related liver disease

**DOI:** 10.3389/fmed.2026.1777280

**Published:** 2026-03-23

**Authors:** Qiufeng He, Fangfang Duan, Chuangjie Mao, Jun Cheng, Yazhi Wang, Yilan Zeng, Song Yang

**Affiliations:** 1 Department of Hepatology, Public Health Clinical Center of Chengdu, Chengdu, Sichuan, China; 2 Department of Hepatology, Beijing Ditan Hospital, Capital Medical University, Beijing, China; 3 Department of Infectious Diseases, China-Japan Friendship Hospital, Beijing, China

**Keywords:** alcohol-related liver disease, prognosis, sarcopenia, spontaneous bacterial peritonitis, vitamin D deficiency

## Abstract

**Background:**

Vitamin D deficiency has been associated with adverse outcomes in various chronic diseases, and its relevance in alcohol-related liver disease (ALD) has attracted increasing attention. This study aimed to evaluate the prognostic significance of vitamin D status in hospitalized patients with ALD.

**Methods:**

We retrospectively analyzed 115 hospitalized patients with ALD between 2021 and 2024. Vitamin D deficiency was defined as serum 25-hydroxyvitamin D [25(OH)D] < 20 ng/mL. Statistical analyses were performed using Spearman’s correlation, logistic regression, Kaplan–Meier survival analysis, multivariable Cox proportional hazards models, and receiver operating characteristic (ROC) curve analysis.

**Results:**

Vitamin D deficiency was present in 35.7% of patients. After adjustment for season, vitamin D deficiency was associated with alcoholic hepatitis, cirrhosis, ascites, sarcopenia, total bilirubin, Model for End-Stage Liver Disease score, and Maddrey discriminant function ≥ 32 (all *p* < 0.05). In multivariable logistic regression, vitamin D deficiency independently predicted greater disease severity (OR 3.087, 95% CI 1.034–9.215; *p* = 0.043). Kaplan–Meier analysis showed reduced survival among patients with vitamin D deficiency (log-rank *p* = 0.025), and multivariable Cox regression confirmed vitamin D deficiency as an independent predictor of mortality (HR 3.179, 95% CI 1.064–9.500; *p* = 0.038). ROC analyses indicated modest discrimination of serum 25(OH)D for sarcopenia and spontaneous bacterial peritonitis (SBP), with optimal cut-offs of 22.74 and 14.2 ng/mL, respectively.

**Conclusion:**

In this cohort of hospitalized ALD patients, vitamin D deficiency was associated with greater disease severity, reduced survival, and increased risk of sarcopenia and SBP. Serum 25(OH)D may serve as a prognostic marker of overall disease burden. Given the retrospective design, limited event numbers, and short follow-up, these findings should be considered exploratory and require confirmation in prospective studies.

## 1 Introduction

Harmful alcohol use is responsible for more than 200 diseases and injuries, with alcohol-related liver disease (ALD) representing one of the most prevalent and clinically consequential manifestations (1). ALD encompasses a broad disease spectrum, ranging from fatty liver and alcoholic hepatitis (AH) to fibrosis, cirrhosis, and hepatocellular carcinoma (HCC) (2). In recent years, ALD has emerged as a leading indication for liver transplantation and accounts for a substantial proportion of liver-related mortality worldwide (3). Despite advances in supportive care, prognosis in ALD remains highly heterogeneous and is influenced by alcohol exposure and abstinence, host susceptibility, comorbidities, and the development of clinical complications.

Vitamin D is a multifunctional steroid hormone best known for its role in bone and mineral metabolism (4). It is hydroxylated in the liver to 25-hydroxyvitamin D [25(OH)D], the principal circulating form and standard biomarker of vitamin D status. Vitamin D deficiency is commonly reported in patients with chronic liver diseases (CLD) and has been associated with advanced fibrosis, cirrhosis, and adverse clinical outcomes, including mortality (5, 6). However, most available evidence derives from viral hepatitis and non-alcoholic fatty liver disease (NAFLD), and evidence specific to ALD remain comparatively limited and inconsistent (7, 8).

Sarcopenia and bacterial infections represent major drivers of morbidity and mortality in advanced liver disease. Sarcopenia, characterized by loss of skeletal muscle mass and function, is highly prevalent in cirrhosis and is associated with impaired quality of life and poor survival (9). Prior studies have reported associations between low serum 25(OH)D levels and sarcopenia in patients with CLD (10, 11). Spontaneous bacterial peritonitis (SBP), a frequent and life-threatening complication of cirrhosis with ascites, has also been linked to vitamin D deficiency in observational studies (12, 13), with limited interventional data suggesting potential benefit of supplementation in selected populations (14). Whether these associations extend to patients with ALD remains unclear.

Accordingly, we investigated the prognostic relevance of vitamin D deficiency in hospitalized patients with ALD, focusing on disease severity, survival, sarcopenia, and SBP. We further explored its potential utility as a clinically accessible risk stratification biomarker.

## 2 Materials and methods

### 2.1 Patients and study design

This retrospective study included 115 consecutive patients with ALD who were hospitalized in the Department of Hepatology at a tertiary hospital in Beijing, China, between January 2021 and April 2024. ALD was diagnosed in accordance with the European Association for the Study of the Liver (EASL) Clinical Practice Guidelines for the Management of Alcohol-Related Liver Disease (15). Patients were excluded if they met any of the following criteria: (1) coexisting viral hepatitis, drug-induced liver injury, autoimmune liver disease, or other chronic liver diseases; (2) prior liver transplantation; (3) use of vitamin D supplements or medications known to affect serum vitamin D levels within the preceding 12 months; or (4) incomplete follow-up data.

**FIGURE 1:**
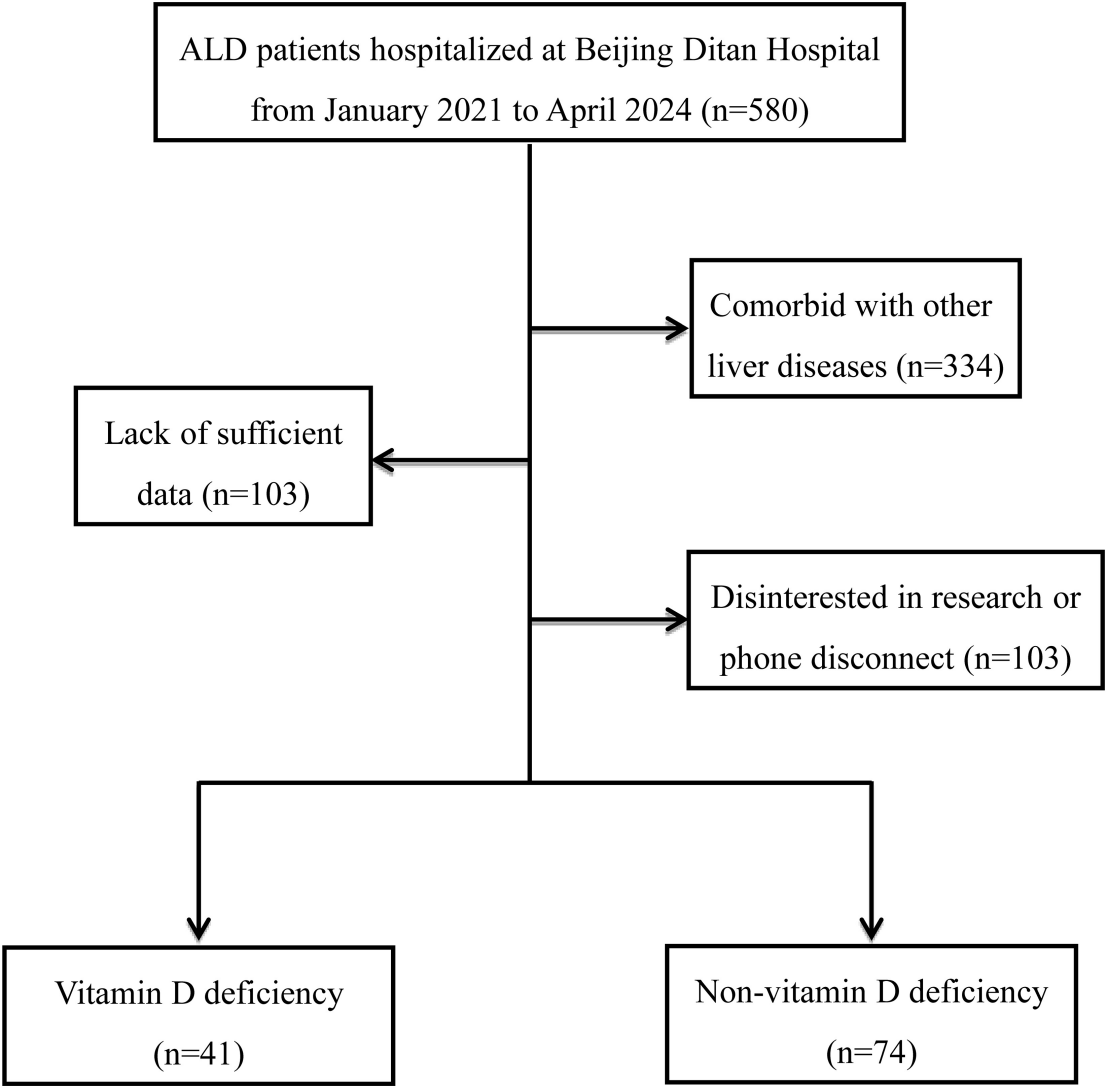
Selection process of the study population.

A total of 580 hospitalized patients with an initial diagnosis of ALD were screened using the hospital information system. Of these, 334 were excluded because of coexisting liver diseases, and 103 were excluded because of insufficient clinical data, leaving 143 patients eligible for further screening. An additional 28 patients were excluded because of inability to be contacted or refusal to participate. Ultimately, 115 patients were included in the final analysis (Figure 1).

The study was approved by the Institutional Ethics Committee of the study hospital, with a waiver of informed consent due to its retrospective design, and was conducted in accordance with the Declaration of Helsinki.

### 2.2 Clinical and laboratory assessment

Baseline demographic and clinical variables included age, sex, season of vitamin D measurement, body mass index (BMI), alcohol abstinence status, and smoking history. Laboratory assessments included complete blood counts, coagulation parameters, liver and renal function tests, serum electrolyte concentrations, and serum 25(OH)D levels. Cirrhosis-related complications, including ascites, SBP, variceal bleeding, hepatic encephalopathy, and HCC, were systematically recorded. All laboratory measurements were performed using standard automated methods in the hospital central laboratory. Serum 25(OH)D levels were measured by chemiluminescence immunoassay (Roche Diagnostics GmbH, Germany).

In accordance with the Central and Eastern European Expert Consensus Statement (16), patients were categorized into a vitamin D deficiency group (<20 ng/mL) and a non-deficiency group (≥20 ng/mL). Consistent with the American Association for the Study of Liver Diseases (AASLD) guidelines (17), SBP was diagnosed as a polymorphonuclear leukocyte count > 250 cells/mmł in ascitic fluid. Liver disease severity was assessed using the Child–Pugh classification, Model for End-Stage Liver Disease (MELD) score, and Maddrey discriminant function (MDF). Patients who were alive at the last contact were censored at the date of last follow-up.

### 2.3 CT scan and assessment of sarcopenia

Computed tomography (CT) imaging is considered a reference standard for objective assessment of body composition in clinical research (18). The skeletal muscle index (SMI) at the level of the third lumbar vertebra (L3) has been validated as a reliable surrogate of whole-body skeletal muscle mass and is widely used for sarcopenia assessment (19). The L3-SMI is expressed as the cross-sectional skeletal muscle area at the L3 level divided by the square of height (cm^2^/m^2^). CT scans were performed using a multi-slice spiral CT scanner (uCT780 256-slice scanner, United Imaging Medical Systems, Shanghai, China). A single axial CT slice at the L3 level was selected for each patient. Skeletal muscle area was quantified using SliceOmatic software (version 5.0, Tomovision, Montreal, Canada). All CT images were independently analyzed by two trained radiologists who were blinded to clinical outcomes.

Given the known influence of ethnicity, lifestyle, and environmental factors on skeletal muscle mass, sarcopenia cut-off values were based on population-specific thresholds (20). According to a prior study in a Chinese population (21), sarcopenia was defined as an L3-SMI < 40.2 cm^2^/m^2^ for men and <31.6 cm^2^/m^2^ for women.

### 2.4 Statistical analysis

Continuous variables are expressed as mean ± standard deviation (SD) or median [interquartile range (IQR)] and were compared using the independent-sample *t*-test or Mann–Whitney U test. Categorical variables are presented as counts and percentages and were compared using the chi-square or Fisher’s exact test. Spearman’s rank correlation analysis was used to assess associations between serum 25(OH)D levels and clinical variables, with seasonal adjustment where applicable. Logistic regression models identified factors associated with disease severity. Cumulative survival was estimated using the Kaplan–Meier method and compared with the log-rank test. Multivariable Cox proportional hazards models were constructed to evaluate independent predictors of mortality. Variables were selected a priori based on clinical relevance and prior literature rather than solely on univariable significance. The proportional hazards assumption was formally evaluated using Schoenfeld residuals, and no significant violations were detected for variables in the final model. Given the limited number of death events, the number of covariates was restricted to reduce overfitting and preserve model stability. Receiver operating characteristic (ROC) curve analyses were performed exploratorily to evaluate the discriminatory performance of serum 25(OH)D for sarcopenia and SBP and to identify potential cut-off values. All analyses were conducted using IBM SPSS Statistics (version 26.0) and R (version 4.3.2). A two-sided *p*-value < 0.05 was considered statistically significant.

## 3 Results

### 3.1 Baseline characteristics

A total of 115 hospitalized patients with ALD were included, with a mean age of 56.4 ± 11.2 years; of whom 108 (93.9%) were male. The overall prevalence of vitamin D deficiency was 35.7%. Serum 25(OH)D levels were 12.80 (8.43–15.40) ng/mL in the deficiency group and 32.52 (28.11–35.11) ng/mL in the non-deficiency group. No significant differences were observed between groups in age, sex, BMI, smoking status, alcohol abstinence, cirrhosis, HCC, variceal bleeding, and hepatic encephalopathy (all *P* > 0.05). Baseline characteristics are summarized in Table 1.

**TABLE 1:** Baseline characteristics of patients with and without vitamin D deficiency.

Variable	Vitamin D deficiency (*n* = 41)	Non-vitamin D deficiency (*n* = 74)	*P-*value
Clinical characteristics			
Age, year	53.88 ± 11.16	57.73 ± 11.25	0.085
Male, *n*%	38 (92.7)	70 (94.6)	0.997
BMI, kg/m^2^	24.21 (21.10–25.71)	23.94 (21.49–26.40)	0.991
Smoking, *n*%	31 (75.6)	56 (75.7)	0.994
Abstinence, *n*%	19 (46.3)	27 (36.5)	0.301
Season^&^, *n*%	12 (29.3)	50 (67.6)	<0.001*
AH, *n*%	21 (51.2)	21 (28.4)	0.015*
Cirrhosis, *n*%	40 (97.6)	64 (86.5)	0.109
HCC, *n*%	5 (12.2)	8 (10.8)	1.000
Ascites, *n*%	36 (87.8)	52 (70.3)	0.034*
SBP, *n*%	14 (34.1)	13 (17.6)	0.045*
Varices bleeding, *n*%	3 (7.3)	3 (4.1)	0.752
Encephalopathy, *n*%	6 (14.6)	9 (12.2)	0.706
Sarcopenia^#^, *n*%	18 (43.9)	18 (24.3)	0.005*
Laboratory parament			
WBC (10^9^/L)	5.76 (4.18–7.50)	4.95 (3.55–6.93)	0.144
HB (g/L)	100.13 ± 29.30	106.93 ± 30.45	0.196
PLT (10^9^/L)	93.00 (50.75–174.25)	112.00 (69.00–168.50)	0.417
INR	1.59 ± 0.45	1.47 ± 0.44	0.093
ALT (U/L)	22.85 (16.80–28.88)	21.00 (13.75–35.45)	0.696
AST (U/L)	41.35 (29.67–71.40)	38.10 (24.65–72.65)	0.337
γ-GT (U/L)	72.10 (33.55–193.80)	70.70 (25.00–175.00)	0.563
TBIL (μmol/L)	46.25 (19.13–109.65)	32.90 (20.85–55.70)	0.198
ALB (g/L)	30.15 (27.92–32.48)	32.15 ± 5.84	0.122
AFP (ng/ml)	3.67 (2.45–6.05)	3.33 (2.40–6.05)	0.981
Cr (μmol/L)	72.45 (63.08–94.68)	70.90 (56.25–84.50)	0.228
Na (mmol/L)	137.80 (134.65–140.75)	137.79 ± 4.84	0.489
Assessment of disease severity			
MELD	16.14 ± 6.09	13.22 (9.73–16.51)	0.043*
MELD ≥ 18, *n*%	17 (41.5)	16 (21.6)	0.024*
MDF	33.29 (21.36–52.92)	23.37 (14.60–36.54)	0.015*
MDF ≥ 32, *n*%	22 (53.7)	23 (31.1)	0.017*
Child-Pugh B/C, *n*%	37 (90.2)	53 (71.6)	0.020*

Data are presented as *n* (%), means ± SD, or median (IQR), respectively. Vitamin D deficiency was defined as serum 25(OH)D < 20 ng/mL. BMI, body mass index; AH, alcoholic hepatitis; HCC, hepatocellular carcinoma; SBP, spontaneous bacterial peritonitis; WBC, white blood cell; HB, hemoglobin; PLT, platelet count; INR, international normalized ratio; ALT, alanine transaminase; AST, aspartate transaminase; γ-GT, γ-glutamyl transpeptidase; TBIL, total bilirubin; ALB, albumin; AFP, alpha-fetoprotein; Cr, creatinine; MELD, model for end-stage liver disease; MDF, Maddrey discriminant function.

^&^Blood sampling during summer/autumn.

^#^Data on sarcopenia missed in 14 patients.

*P-value < 0.05 was considered significant.

### 3.2 Factors associated with vitamin D deficiency

Correlations between vitamin D deficiency and clinical variables are presented in Table 2. Given the known seasonal variation in serum 25(OH)D, Spearman’s correlation analyses were performed both before and after adjustment for season. In unadjusted analyses, vitamin D deficiency correlated positively with AH, ascites, SBP, sarcopenia, total bilirubin (TBIL), MELD score, MDF, and Child–Pugh B/C (all *p* < 0.05). After adjustment for season, significant positive correlations persisted for AH (*r* = 0.207, *p* = 0.027), cirrhosis (*r* = 0.241, *p* = 0.010), ascites (*r* = 0.190, *p* = 0.043), sarcopenia (*r* = 0.213, *p* = 0.033), TBIL (*r* = 0.212, *p* = 0.024), MELD score (*r* = 0.192, *p* = 0.040), and MDF ≥ 32 (*r* = 0.207, *p* = 0.027).

**TABLE 2:** Factors associated with vitamin D deficiency in patients with alcohol-related liver disease (ALD).

Characteristic	unadjusted		Adjusted^a^	
	*r*	*P-*value	*r*	*P-*value
Age	−0.162	0.085	−0.180	0.055
Male	−0.038	0.684	−0.048	0.614
BMI	−0.014	0.879	−0.03	0.975
AH	0.227	0.015*	0.207	0.027*
Cirrhosis	0.180	0.054	0.241	0.010*
Ascites	0.198	0.034*	0.190	0.043*
SBP	0.187	0.045*	0.130	0.169
Sarcopenia^#^	0.275	0.005*	0.213	0.033*
PLT	0.093	0.323	0.017	0.858
INR	0.157	0.093	0.132	0.160
ALT	−0.069	0.467	−0.063	0.505
AST	0.054	0.569	0.043	0.652
TBIL	0.220	0.018*	0.212	0.024*
Cr	0.048	0.612	0.036	0.706
Na	0.007	0.945	0.003	0.977
MELD	0.219	0.019*	0.192	0.040*
MELD ≥ 18	0.210	0.024*	0.170	0.071
MDF	0.203	0.029*	0.173	0.066
MDF ≥ 32	0.222	0.017*	0.207	0.027*
Child-Pugh B/C	0.216	0.020*	0.176	0.062

*^a^*Adjusted for season.

^#^Data on sarcopenia missed in 14 patients.

*P-value < 0.05 was considered significant. BMI, body mass index; AH, alcoholic hepatitis; SBP, spontaneous bacterial peritonitis; PLT, platelet count; INR, international normalized ratio; ALT, alanine transaminase; AST, aspartate transaminase; TBIL, total bilirubin; Cr, creatinine; MELD, model for end-stage liver disease; MDF, Maddrey discriminant function.

### 3.3 Logistic regression analysis of ALD severity

Univariate logistic regression identified several factors significantly associated with greater ALD severity, including age (OR 0.940, 95% CI 0.905–0.977; *p* = 0.002), ascites (OR 3.667, 95% CI 1.273–10.560; *p* = 0.016), vitamin D deficiency (OR 2.568, 95% CI 1.169–5.639; *p* = 0.019), hemoglobin (HB) (OR 0.970, 95% CI 0.955–0.985; *p* < 0.001), platelet count (PLT) (OR 0.990, 95% CI 0.984–0.996; *p* = 0.002), and albumin (ALB) (OR 0.823, 95% CI 0.746–0.908; *p* < 0.001).

In multivariate analysis, age (OR 0.923, 95% CI 0.878–0.970; *p* = 0.002), vitamin D deficiency (OR 3.087, 95% CI 1.034–9.215; *p* = 0.043), HB (OR 0.976, 95% CI 0.956–0.996; *p* = 0.020), PLT (OR 0.989, 95% CI 0.981–0.998; *p* = 0.011), and ALB (OR 0.836, 95% CI 0.735–0.950; *p* = 0.006) remained independently associated with ALD severity. Detailed results are shown in Table 3.

**TABLE 3:** Logistic regression analysis of alcohol-related liver disease (ALD) severity.

Variables	Univariable analysis			Multivariable analysis		
	OR	95% CI	*P*-value	OR	95% CI	*P-*value
Age	0.940	0.905–0.977	0.002*	0.923	0.878–0.970	0.002*
Male	0.235	0.044–1.270	0.093	–	–	–
BMI	1.009	0.923–1.103	0.849	–	–	–
Season^&^	0.717	0.338–1.522	0.387	–	–	–
Abstinence	1.164	0.543–2.495	0.697	–	–	–
AH	1.093	0.503–2.375	0.823	–	–	–
HCC	0.429	0.111–1.652	0.218	–	–	–
Ascites	3.667	1.273–10.560	0.016*	0.834	0.192–3.620	0.809
SBP	1.333	0.557–3.193	0.518	–	–	–
Sarcopenia^#^	1.564	0.679–3.601	0.294	–	–	–
Vitamin D deficiency	2.568	1.169–5.639	0.019*	3.087	1.034–9.215	0.043*
HB	0.970	0.955–0.985	<0.001*	0.976	0.956–0.996	0.020*
PLT	0.990	0.984–0.996	0.002*	0.989	0.981–0.998	0.011*
ALT	0.996	0.987–1.006	0.440	–	–	–
AST	1.002	0.997–1.007	0.497	–	–	–
γ-GT	0.996	0.992–0.999	0.010	–	–	–
ALB	0.823	0.746–0.908	<0.001*	0.836	0.735–0.950	0.006*
Cr	1.000	0.993–1.007	0.973	–	–	–
Na	0.932	0.855–1.016	0.932	–	–	–

^&^Blood sampling during summer/autumn.

^#^Data on sarcopenia missed in 14 patients.

*P-value < 0.05 was considered significant. OR, odds ratio; CI, confidence interval; BMI, body mass index; AH, alcoholic hepatitis; HCC, hepatocellular carcinoma; SBP, spontaneous bacterial peritonitis; HB, hemoglobin; PLT, platelet count; ALT, alanine transaminase; AST, aspartate transaminase; γ-GT, γ-glutamyl transpeptidase; ALB, albumin; Cr, creatinine.

### 3.4 Kaplan–Meier survival and Cox regression analyses

**FIGURE 2:**
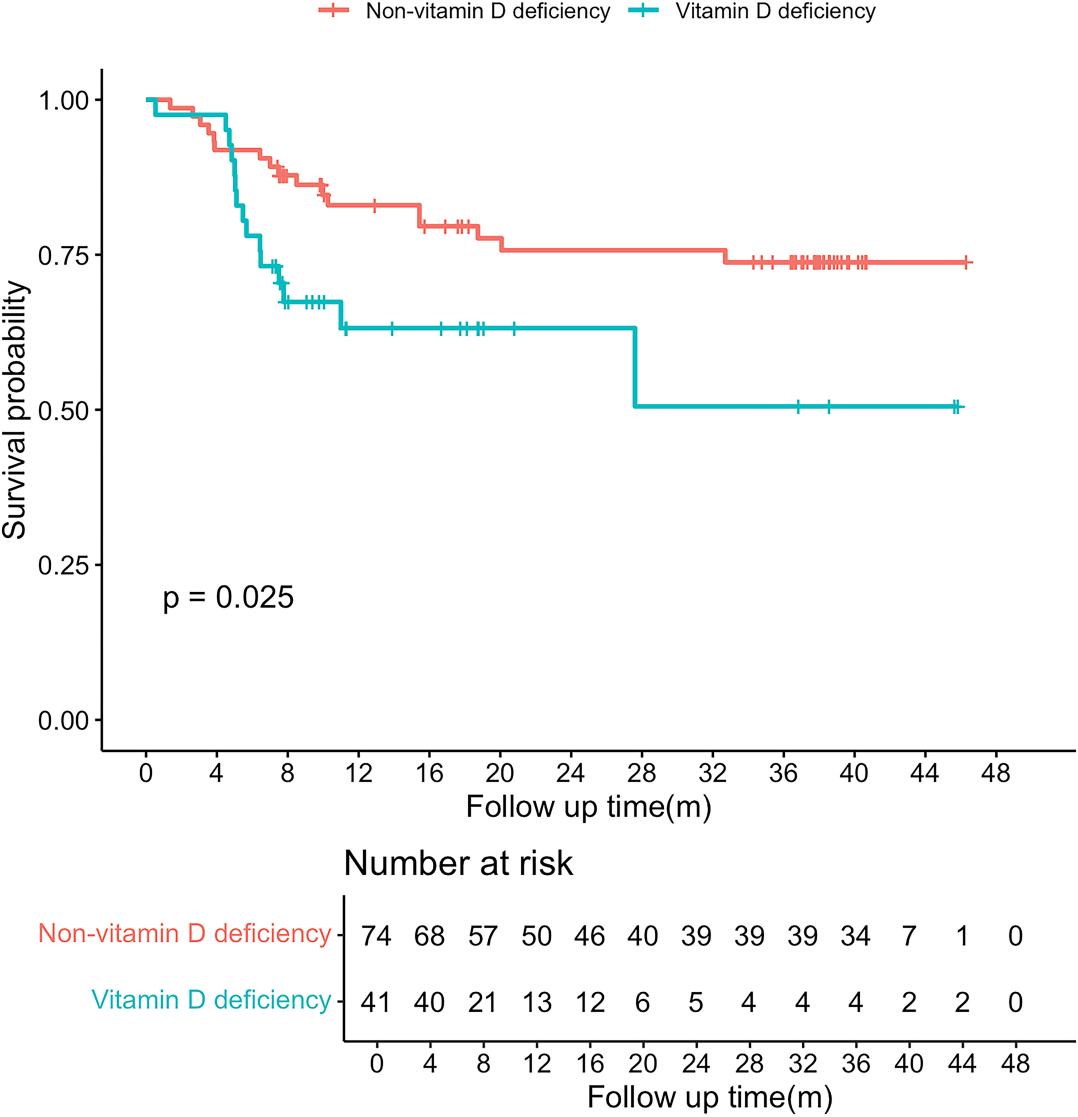
Comparison of cumulative survival rates in alcohol-related liver disease (ALD) patients with and without vitamin D deficiency.

The median follow-up duration was 9.25 months (7.21–17.63), during which 32 patients (27.8%) died from liver disease–related causes. Kaplan–Meier analysis showed significantly reduced survival in patients with vitamin D deficiency compared with those without deficiency (log-rank *p* = 0.025; Figure 2).

Univariable Cox analysis identified AH, SBP, white blood cell count (WBC), HB, γ-glutamyl transpeptidase (γ-GT), ALB, creatinine (Cr), sodium and MELD score were associated with mortality (Supplementary Table 1). In multivariable models adjusting for key demographic and disease-related factors, vitamin D deficiency remained independently associated with mortality (HR 3.179, 95% CI 1.064–9.500; *p* = 0.038). serum Cr (HR 1.007, 95% CI 1.003–1.011; *p* < 0.001) and sodium (HR 0.866, 95% CI 0.794–0.945; *p* = 0.001) were also independent predictors of mortality.

### 3.5 ROC curve analysis of 25(OH)D for sarcopenia and SBP

**FIGURE 3:**
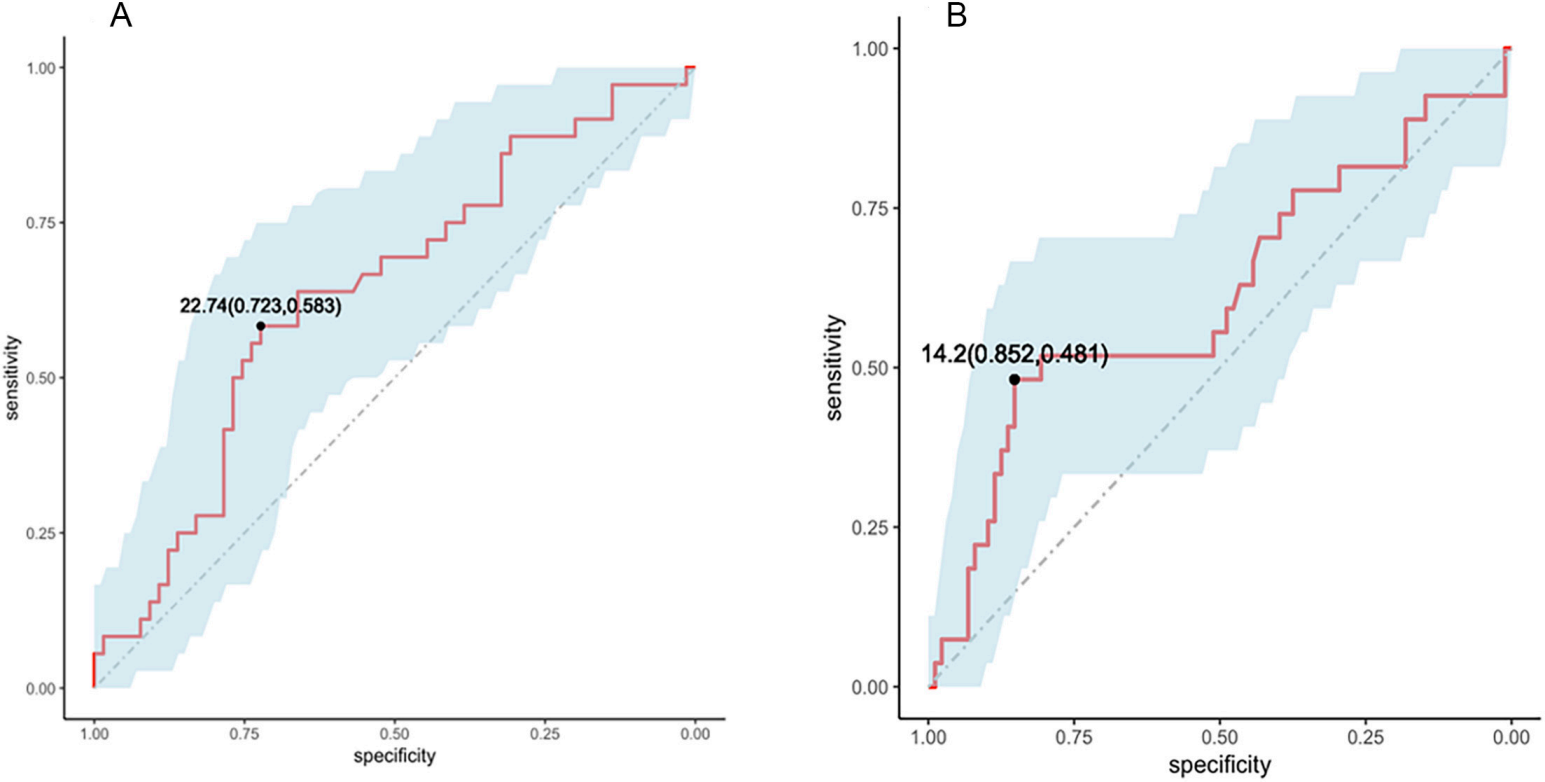
Receiver operating characteristic (ROC) curve analysis of serum 25(OH)D levels for sarcopenia **(A)** and spontaneous bacterial peritonitis (SBP) **(B)** in alcohol-related liver disease (ALD) patients. The red curve in the figure represents the ROC curve, and the shaded area represents the 95% confidence interval.

Receiver operating characteristic curve analyses evaluated the ability of serum 25(OH)D to discriminate sarcopenia and SBP in patients with ALD (Figure 3). For sarcopenia, the area under the ROC curve (AUC) was 0.640 (95% CI 0.526–0.753). A cut-off value of 22.74 ng/mL yielded a sensitivity of 58.3% and specificity of 72.3%. For SBP, the AUC was 0.613 (95% CI 0.479–0.747), with a cut-off of 14.2 ng/mL corresponding to 48.1% sensitivity and 85.2% specificity.

## 4 Discussion

In this retrospective cohort of hospitalized patients with ALD, vitamin D deficiency was common and associated with adverse clinical outcomes. Lower 25(OH)D levels were linked to greater disease severity, reduced survival, sarcopenia, and SBP. Although vitamin D has been extensively studied in NAFLD and chronic viral hepatitis, evidence concerning its association with ALD remains relatively limited. To our knowledge, this study provides a comprehensive evaluation of these associations in a Chinese ALD cohort. However, given the retrospective, single-centre design, these findings should be interpreted as associative rather than causal.

The relationship between vitamin D status and liver dysfunction is likely bidirectional. Advanced liver disease may reduce circulating 25(OH)D levels through impaired hepatic hydroxylation, reduced synthesis of vitamin D-binding protein, malabsorption, and systemic inflammation. Conversely, vitamin D deficiency may exacerbate hepatic injury via attenuation of anti-inflammatory, anti-fibrotic, and immunomodulatory pathways, thereby promoting inflammation and fibrogenesis (22, 23). Within this framework, low serum 25(OH)D may function both as a marker of advanced disease and as a potential modifier of disease progression. Accordingly, reduced vitamin D levels may primarily reflect disease severity, systemic inflammation, and nutritional impairment rather than act as an independent causal determinant of outcomes.

Consistent with prior reports, vitamin D deficiency was common in our cohort. Previous studies in CLD have reported deficiency rates ranging from 64% to 92% (24), and similarly high rates have been observed among individuals with alcohol use disorder in Asia and Europe (25–27). In the present study, more than one-third of ALD patients were deficient, and fewer than half had sufficient vitamin D levels. This high prevalence likely reflects multiple contributing mechanisms, including inadequate intake, impaired intestinal absorption, reduced sunlight exposure, diminished hepatic hydroxylation, decreased vitamin D–binding protein synthesis, and chronic inflammation (28). Experimental studies further indicate that active vitamin D metabolites may attenuate alcohol-induced hepatic inflammation, supporting biological plausibility for an association with disease severity (29).

Although cutaneous synthesis is the principal source of vitamin D, a single-timepoint measurement may not reflect long-term status (30). Circulating 25(OH)D levels are influenced by acute illness, systemic inflammation, reduced mobility, malnutrition, and impaired hepatic metabolism. Thus, serum 25(OH)D likely represents an integrated signal of nutritional, metabolic, inflammatory, and liver disease. Despite adjustment for seasonal variation, residual confounding is likely. Our findings are concordant with prior studies linking vitamin D deficiency to fibrosis and cirrhosis in chronic hepatitis C and NAFLD (31, 32). We also observed associations with AH and elevated TBIL, consistent with previous reports connecting low 25(OH)D levels with greater disease severity and hyperbilirubinemia in CLD (33, 34).

Vitamin D deficiency was also associated with established indices of disease severity. Prior studies (35–37) have reported inverse associations between serum 25(OH)D levels and both Child–Pugh and MELD score. We additionally incorporated the MDF, given its established prognostic relevance in alcoholic hepatitis (38). Consistent with previous reports, lower 25(OH)D levels in our cohort were associated with more advanced disease severity ALD. Other independently associated variables, including age, PLT, ALB, and HB, are well-recognized markers reflecting hepatic fibrosis, portal hypertension, nutritional status, and overall systemic disease burden. The associations observed for age and PLT may partly reflect underlying fibrosis, as captured by composite indices such as FIB-4 (39). Together, these concordant findings support the robustness of the observed associations.

The association between vitamin D status and survival in liver disease remains controversial. While some studies have reported attenuation of this association after adjustment for disease severity, others have observed a relationship between low 25(OH)D levels and mortality in cirrhosis (40–42). In our cohort, vitamin D deficiency was associated with reduced survival in Kaplan–Meier analyses and remained independently associated with mortality after multivariable adjustment; however, the hazard ratio was accompanied by a relatively wide confidence interval, reflecting limited precision due to the modest number of death events and relatively short follow-up duration. The modest number of deaths and the short median follow-up likely reduce the stability of survival estimates, particularly at longer time points (e.g., 24- and 36-month survival). In addition, censoring patterns and loss to follow-up were not systematically assessed and may have influenced survival estimates. Therefore, serum 25(OH)D should be regarded primarily as a prognostic marker of overall disease burden rather than as a proven causal determinant of mortality. These findings should be considered exploratory and require confirmation in larger prospective cohorts with adequate event numbers and longer follow-up.

The association between vitamin D deficiency and sarcopenia represents a clinically relevant finding. In our cohort, 31.3% of hospitalized patients with ALD had sarcopenia, consistent with prior reports in Chinese and ALD populations (21, 43). Sarcopenia was more prevalent among patients with vitamin D deficiency, in line with previous studies in CLD linking low 25(OH)D levels to reduced skeletal muscle mass and function (44–46). However, data specific to ALD remain limited. ROC curve analyses demonstrated modest discrimination of serum 25(OH)D for sarcopenia, with an optimal cut-off value of 22.74 ng/mL. Given the limited sample size and number of events, these findings should be considered exploratory. Vitamin D status may therefore serve as a complementary biomarker for identifying patients at higher risk of reduced muscle mass rather than as a standalone diagnostic tool for sarcopenia. Mechanistically, active vitamin D metabolites are involved in muscle contraction, cellular proliferation, and differentiation, and deficiency has been associated with muscle mass loss and impaired function (47–49), supporting biological plausibility. Notably, sarcopenia in the present study was defined solely by CT-derived L3-SMI. Current consensus definitions, including those from the European Working Group on Sarcopenia in Older People (EWGSOP2) and the Asian Working Group for Sarcopenia (AWGS), require both low muscle mass and impaired muscle strength and/or performance (50, 51). Reliance on muscle mass alone may therefore overestimate or misclassify clinical sarcopenia. Our findings should thus be interpreted as reflecting reduced skeletal muscle mass rather than fully defined sarcopenia. Prospective studies incorporating both structural and functional assessments are needed to clarify the role of vitamin D in sarcopenia among patients with ALD.

We also observed an association between vitamin D deficiency and SBP. Previous studies in cirrhosis and chronic hepatitis C have linked low 25(OH)D levels to increased susceptibility to bacterial infections (13, 40). In our cohort, ROC analysis demonstrated modest discrimination of serum 25(OH)D for SBP, with a cut-off of 14.2 ng/mL characterized by high specificity but limited sensitivity. These results suggest that low vitamin D levels may help identify patients at increased risk for SBP, but should not be interpreted as a definitive diagnostic or screening test. The mechanisms linking vitamin D deficiency to infection risk in advanced liver disease are not fully understood. Potential mechanisms include impaired innate immune responses, altered toll-like receptor signaling, and compromised gut barrier integrity facilitating bacterial translocation (52–54). Given the limited number of SBP events, serum 25(OH)D should be interpreted primarily as a marker of infection susceptibility rather than a causal determinant of SBP.

This study has several limitations. First, the retrospective, single-center design precludes causal inference and is susceptible to selection bias, residual confounding, and reverse causality. Advanced liver dysfunction itself may reduce circulating 25(OH)D levels through impaired hepatic hydroxylation, decreased vitamin D–binding protein synthesis, and systemic inflammation, such that low vitamin D levels may partly reflect underlying disease severity rather than a direct causal effect. Second, the modest sample size and limited numbers of key outcome events, including deaths, sarcopenia, and SBP, may reduce statistical power and increase the risk of model instability. Although multivariable logistic and Cox regression models were performed, the relatively small number of events and short follow-up duration restrict the robustness and precision of the effect estimates, and residual confounding by disease severity and related prognostic factors cannot be excluded. While the number of covariates included in the multivariable Cox model was deliberately restricted relative to the event count to mitigate overfitting, the modest number of deaths may still compromise model stability and the reliability of survival estimates. Third, vitamin D was measured at a single time point and may reflect acute illness, inflammatory status, and nutritional impairment rather than long-term exposure. Although seasonal variation was adjusted for and supplement users were excluded, other determinants, including dietary intake, sunlight exposure, and physical activity, were not systematically captured, leaving potential residual confounding. In addition, the relatively narrow IQR observed for serum 25(OH)D levels likely reflects the relatively homogeneous inpatient population and laboratory measurement timing. Fourth, sarcopenia was defined solely by CT-derived muscle mass without assessment of muscle strength or physical performance. According to contemporary consensus definitions, this approach may not fully capture multidimensional sarcopenia and should be interpreted as reflecting reduced skeletal muscle mass. Finally, the cohort was predominantly male, limiting generalizability to female patients and potentially affecting interpretation of body composition–related findings. Given known sex-specific differences in muscle mass, hormonal regulation, and vitamin D metabolism, extrapolation of these findings to women should be made with caution. Future multicenter, prospective studies incorporating longitudinal vitamin D assessment, functional sarcopenia measures, and larger event numbers are needed to clarify the independent and potentially causal role of vitamin D in ALD.

## 5 Conclusion

In conclusion, vitamin D deficiency was common among hospitalized patients with ALD and was consistently associated with greater disease severity, reduced survival, and increased risk of sarcopenia and SBP. These findings suggest that serum 25(OH)D may serve as a clinically accessible biomarker reflecting overall disease burden and susceptibility to complications. However, given the retrospective design and the potential for reverse causality and residual confounding, these associations should be interpreted as prognostic rather than causal. Whether correction of vitamin D deficiency improves clinical outcomes in ALD remains uncertain. Large, multicenter prospective studies and randomized trials are required to clarify the independent and potentially modifiable role of vitamin D in ALD progression and its complications.

## Data Availability

The raw data supporting the conclusions of this article will be made available by the authors, without undue reservation.
