# Supplementary material for: Prognostic associations of vitamin D deficiency with disease severity, survival, and complications in alcohol-related liver disease
Source: Front Med (Lausanne). 2026 Mar 23;13:1777280. doi: 10.3389/fmed.2026.1777280 (PMC13050956; doi:10.3389/fmed.2026.1777280)
Supplement: Supplementary file 1 [file Data_Sheet_1.pdf]

Supplementary Table 1. Cox analysis of survival in patients with ALD.

Variables	Univariable analysis			Multivariable analysis		
	HR	95% CI	P value	HR	95% CI	P value
Age, year	1.039	1.000-1.079	0.052	1.039	0.991-1.089	0.109
Male, n%	1.288	0.172-9.628	0.805			
BMI, kg/m^2	1.035	0.938-1.141	0.493			
Smoking, n%	0.414	0.171-1.000	0.050			
Abstinence, n%	0.758	0.303-1.901	0.555	0.894	0.371-2.155	0.802
Season[&], n%	1.226	0.468-3.210	0.679	2.145	0.688-6.687	0.188
AH, n%	5.639	2.011-15.813	0.001*			
Cirrhosis, n%	22.466	0.010-48754.61	0.427			
HCC, n%	1.221	0.283-5.275	0.789			
Ascites, n%	6.199	0.830-46.318	0.075			
SBP, n%	3.991	1.653-9.638	0.002*			
Varices bleeding, n%	0.771	0.103-5.764	0.800			
Hepatic encephalopathy, n%	1.021	0.299-3.487	0.974			
Sarcopenia[#], n%	0.688	0.245-1.929	0.477			
Vitamin D deficiency, n%	1.817	0.773-4.479	0.194	3.179	1.064-9.500	0.038*
WBC (10^9/L)	1.144	1.005-1.303	0.042*	1.010	0.857-1.191	0.904
HB (g/L)	0.984	0.971-0.998	0.024*			
PLT (10^9/L)	1.002	0.997-1.007	0.487			
INR	1.454	0.682-3.103	0.333			
ALT (U/L)	0.998	0.990-1.006	0.615			
AST (U/L)	0.999	0.993-1.005	0.714			
γ-GT (U/L)	0.995	0.990-1.000	0.035*			
TBIL (μmol/L)	1.002	0.988-1.007	0.240			
ALB (g/L)	0.886	0.818-0.960	0.003*			
AFP, (ng/ml)	0.955	0.834-1.093	0.504			
Cr (μmol/L)	1.006	1.003-1.009	<0.001*	1.007	1.003-1.011	<0.001*

Na, (mmol/L)	0.869	0.807-0.935	<0.001*	0.866	0.794-0.945	0.001*
MELD	1.080	1.012-1.153	0.020*			
MDF	1.007	0.993-1.020	0.356			
Child-Pugh B/C	6.736	0.908-49.977	0.062			

Note: The final multivariable Cox model included seven covariates (age, abstinence, season, vitamin D deficiency, WBC, Cr, and sodium) for 32 death events. Data are presented as n (%), means ± SD, or median (IQR), respectively. Vitamin D deficiency was defined as serum 25(OH)D < 20 ng/mL. *P value < 0.05 was considered significant. &, blood sampling during summer/autumn. #, data on sarcopenia were missing in 14 patients.

Abbreviations: BMI, body mass index; AH, alcoholic hepatitis; HCC, hepatocellular carcinoma; SBP, spontaneous bacterial peritonitis; WBC, white blood cell; HB, haemoglobin; PLT, platelet count; INR, international normalized ratio; ALT, alanine aminotransferase; AST, aspartate aminotransferase; γ-GT, γ-glutamyl transpeptidase; TBIL, total bilirubin; ALB, albumin; AFP, alpha-fetoprotein; Cr, creatinine; MELD, model for end-stage liver disease; MDF, Maddrey discriminant function.
